# Lifestyle Medicine and the Science of Cholesterol Reduction: A Systematic Review of Non-medication Approaches Among Adults

**DOI:** 10.7759/cureus.115435

**Published:** 2026-08-30

**Authors:** Theresa O Akewe, Opeyemi Taiwo, Toluwalope F Ejiyooye, Kosisochi E Achara, Oluwatobi Adebiyi, Ezinwanne J Okoye

**Affiliations:** 1 Family Medicine, University of Benin, Benin City, NGA; 2 Primary Care, Olabisi Onabanjo University, Ago Iwoye, NGA; 3 Family Medicine, Defense Health Agency, San Antonio, USA; 4 Public Health, Emory University, Atlanta, USA; 5 General Medicine, College of Medicine, University of Lagos, Lagos, NGA; 6 Internal Medicine, Chukwuemeka Odumegwu Ojukwu University Teaching Hospital, Awka, NGA

**Keywords:** cardiovascular disease, cholesterol reduction, dyslipidemia, lifestyle medicine, lipid profile, non-pharmacological interventions

## Abstract

Dyslipidemia is a major modifiable risk factor for cardiovascular disease and remains highly prevalent despite advances in lipid-lowering therapy. Lifestyle medicine has emerged as a comprehensive, non-pharmacological strategy for improving lipid profiles and reducing cardiovascular risk. This systematic review was conducted according to the Preferred Reporting Items for Systematic Reviews and Meta-Analyses (PRISMA) 2020 guidelines and evaluated the effectiveness of lifestyle-based interventions for cholesterol reduction in adults. PubMed, Scopus, Web of Science, Embase, and the Cochrane Library were searched for observational studies published between 2015 and May 2026. Studies involving adults (≥18 years) that evaluated lifestyle interventions and reported lipid-related outcomes were included. Study quality was assessed using the Newcastle-Ottawa Scale. Twenty-six studies met the inclusion criteria. Mediterranean and plant-based diets, together with regular physical activity, were consistently associated with reductions in low-density lipoprotein cholesterol and triglycerides, improvements in high-density lipoprotein cholesterol, and lower cardiovascular risk. Weight management, smoking cessation, adequate sleep, and stress reduction were also associated with favorable lipid outcomes. However, long-term adherence was frequently limited by socioeconomic and behavioral barriers. Lifestyle medicine provides an important foundation for dyslipidemia management and should be integrated with pharmacological therapy when clinically indicated. Future research should focus on improving long-term adherence and equitable access to lifestyle-based interventions.

## Introduction and background

Cardiovascular disease (CVD) remains the leading cause of death globally, with high blood cholesterol representing one of the most important modifiable cardiovascular risk factors [1,2]. Atherogenic lipoproteins are important risk factors for atherosclerosis, coronary artery disease, stroke, and premature cardiovascular death. Elevated total cholesterol, low-density lipoprotein cholesterol (LDL-C), and triglyceride levels, together with reduced high-density lipoprotein cholesterol (HDL-C), are important lipid abnormalities identified through the standard lipid profile that are associated with an increased risk of CVD [3]. Cholesterol reduction remains an important public health priority because a substantial proportion of adults in the United States have elevated LDL-C levels or require lipid-lowering treatment [4]. Although lipid-lowering medications, particularly statins, have substantially reduced cardiovascular morbidity and mortality, contemporary dyslipidemia management increasingly emphasizes lifestyle-based interventions, including dietary modification, regular physical activity, weight management, and other evidence-based behavioral strategies that complement pharmacological treatment [5,6].

Lifestyle medicine is based on an evidence-based nature and involves the implementation of therapeutic lifestyle changes to prevent, treat, and in some cases reverse various chronic diseases [7]. Lifestyle medicine has several core principles that include healthy eating habits, engaging in physical activities, keeping normal weight, getting sufficient sleep, coping with stressors, quitting smoking, and moderating alcohol consumption in accordance with established public health recommendations [8,9]. Lifestyle modification remains the foundation of dyslipidemia management and is strongly supported by current clinical guidelines. Evidence-based interventions, including dietary modification, regular physical activity, weight management, smoking cessation, and adherence to healthy lifestyle practices, are recommended for all individuals regardless of their cardiovascular risk profile. Pharmacological treatment decisions should be based on an individual's overall atherosclerotic CVD risk, LDL-C levels, diabetes status, and other cardiovascular risk factors. Statins and other lipid-lowering therapies remain essential because of their well-established benefits in reducing cardiovascular events, while lifestyle interventions serve as complementary strategies within comprehensive cardiovascular risk management programs [6,10].

Lifestyle factors affecting the effectiveness of lifestyle changes are influenced by multiple biological, behavioral, and environmental factors [11]. Evidently, modern dietary eating practices such as the Mediterranean diet, DASH (Dietary Approaches to Stop Hypertension) diet, vegetarian diets, and diets containing large amounts of soluble fibers, whole grains, nuts, legumes, fruits, and vegetables are consistently associated with decreased LDL-C and improved lipid profiles [12]. Regular aerobic and resistance exercises help improve lipid metabolism by establishing higher levels of HDL-C, lower amounts of triglycerides, increased insulin sensitivity, and weight loss. There is convincing evidence that consistent weight loss, effective stress management, and smoking cessation contribute to improved cholesterol levels and overall cardiovascular health [13].

While substantial evidence supports dietary modification as an effective strategy for improving cholesterol levels, maintaining long-term adherence to healthy dietary practices remains a significant challenge [14]. Different issues such as motivation on the part of a person, background, level of education, and access to fresh food, food insecurity, and poor knowledge of the different diets greatly affect the effectiveness of lifestyle changes [15]. Additionally, barriers in access to medical assistance, different social practices in society, and lack of means of socialization lead to differences in attitude toward lifestyle changes [14,15]. There are different approaches suggested through health interventions, which include a multidisciplinary approach, teamwork, and the need for a combination of personalized coaching programs [16]. As dyslipidemia continues to grow, coupled with the need for preventive care in this field, it is critical to look for information about the non-pharmacologic management of cholesterol disorders [17,18].

This systematic review aimed to evaluate the associations between lifestyle medicine interventions and lipid outcomes among adults aged 18 years and older. Specifically, the review examined dietary modification, physical activity, weight management, smoking cessation, sleep optimization, and stress management in relation to changes in LDL-C, HDL-C, triglycerides, total cholesterol, and overall lipid profiles. The review also aimed to identify evidence relevant to clinical practice and highlight areas requiring further research in lifestyle-based dyslipidemia management.

## Review

Materials and methods

Eligibility Criteria and Search Strategies

This systematic review was conducted according to the Preferred Reporting Items for Systematic Reviews and Meta-Analyses (PRISMA) 2020 guidelines to ensure methodological transparency, reproducibility, and rigor [19]. Studies published between January 2015 and May 2026 were considered eligible for inclusion. The literature search strategy was developed using the Population, Intervention, Comparison, and Outcome (PICO) framework [20].

A comprehensive literature search was conducted in PubMed, Scopus, Embase, Web of Science, and the Cochrane Library. Search strategies incorporated combinations of controlled vocabulary terms (including MeSH (Medical Subject Headings) terms, when applicable) and free-text keywords related to lifestyle medicine, dyslipidemia, hypercholesterolemia, cholesterol reduction, lipid profile, dietary intervention, and physical activity. The search strategy was adapted for each database, and the final search was completed in May 2026. Detailed database-specific search strategies, including search terms, search fields, and applied filters, are provided in the Appendices and summarized in Table 1.

**Table 1:** Overview of search strategy

Category	Details
Databases Searched	PubMed, Scopus, Web of Science, Embase, and the Cochrane Library
Final Search Date	May 2026
Time Frame	Studies published between January 2015 and May 2026
Language	English only
Search Strategy Structure	The search strategy was developed using the PICO framework and a combination of controlled vocabulary terms and free-text keywords
#1 (Population)	Adult population OR adults OR men OR women
#2 (Exposure/Risk Factors/Intervention)	Lifestyle medicine OR lifestyle intervention OR dietary intervention OR Mediterranean diet OR DASH diet OR plant-based diet OR physical activity OR exercise
#3 (Study Context)	United States OR U.S. population
#4 (Outcome)	Cholesterol reduction OR dyslipidemia OR hypercholesterolemia OR lipid profile OR low-density lipoprotein cholesterol (LDL-C) OR high-density lipoprotein cholesterol (HDL-C)

Eligibility Criteria

Eligible studies included adults aged 18 years and older with dyslipidemia, defined as abnormalities in one or more lipid parameters, including hypercholesterolemia, elevated LDL-C, elevated triglyceride levels, or reduced HDL-C, as determined by physician diagnosis, laboratory measurements, or the diagnostic criteria used in the original studies. Studies conducted in the United States were selected specifically to ensure similarity in terms of health care provision, clinical guidelines, and the population. Among the interventions offered in the studies, healthy eating and good nutrition (Mediterranean and DASH diets, etc.), physical exercise, weight management, quitting smoking, sleeping properly, stress management, and others were also examined. Studies had to be observational studies published between 2015 and May 2026. The year 2015 was selected as the starting point to ensure that the review captured contemporary evidence reflecting current lifestyle medicine approaches, cardiovascular prevention strategies, and recent developments in dyslipidemia management. The studies selected must report on the quantitative lipid endpoints, which include changes in LDL-C, HDL-C, triglycerides, and total cardiovascular impact.

Study Selection Process

Once all articles were retrieved, they were organized using reference software to eliminate any duplicates. Two investigators independently screened the titles and abstracts of all retrieved studies to identify potentially eligible articles. Subsequently, both investigators independently assessed the full-text articles for eligibility according to the predefined inclusion and exclusion criteria. Any disagreements regarding study selection were resolved through discussion and consensus.

Quality Assessment

The methodological quality of the included studies was assessed using the Newcastle-Ottawa Scale (NOS) for observational studies [21]. Each study was assigned a risk of bias score according to the quality of selection, comparability, and outcome assessment.

Data Extraction

A standard format for data collection was constructed prior to the collection of data. The variables extracted included author’s name, year of publication, study type, study location, subject characteristics, sample size, type of lifestyle medicine intervention, follow-up duration, lipid outcomes (including LDL-C, HDL-C, total cholesterol, triglycerides), cardiovascular outcomes, adherence measures, and key results. Other parameters included socio-economic factors, obstacles to lifestyle adherence, multi-disciplinary approaches, and digital health solutions if relevant. Two reviewers who could sort out any discrepancies through a deliberation process obtained data independently.

Results

The results of the search yielded 585 records upon performing the search using the mentioned database. After removing duplicates, there remained a total of 475 titles and abstracts, which underwent further screening. Then, 335 articles were removed from the collection because of the exclusion after abstract screening. Specifically, articles that did not address the topic of lifestyle medicine, studies on non-pharmaceutical management of cholesterol, studies carried out outside the borders of the United States, non-human population studies, non-English studies, and those that did not report any findings concerning lipid outcomes were excluded.

In total, 140 full-text articles were then screened for eligibility. Following a detailed full-text review, 114 articles were excluded because they did not meet the predefined eligibility criteria. In the majority of the papers, some methodological issues, improper design of the studies, lack of sufficient reporting on the outcomes in the case of cholesterol and lipid profile analysis, failure to confirm adherence to lifestyle medicine practices, and unreported results were considered reasons to exclude the studies based on the lack of relevant research. After this, 26 articles were found eligible to be used in this study. Figure 1 presents a PRISMA diagram which shows the study selection and inclusion process.

**Figure 1:**
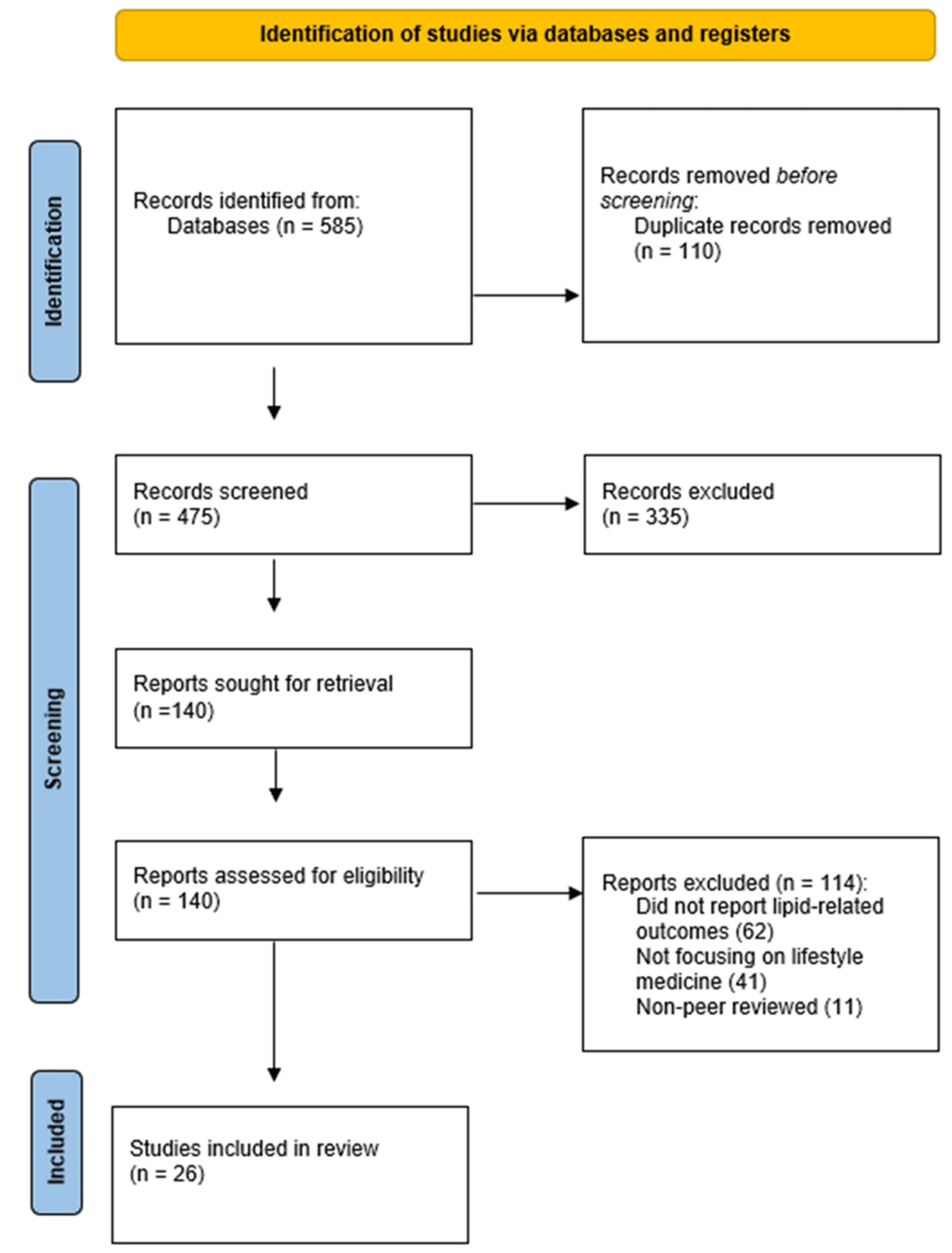
PRISMA flow diagram indicating the study selection and inclusion process PRISMA, Preferred Reporting Items for Systematic Reviews and Meta-Analyses

Table 2 presents a summary of the characteristics of the 26 included studies in this study.

**Table 2:** Summary of the characteristics of included studies ARIC, Atherosclerosis Risk in Communities; CARDIA, Coronary Artery Risk Development in Young Adults; CHD, coronary heart disease; CVD, cardiovascular disease; HDL-C, high-density lipoprotein cholesterol; HPFS, Health Professionals Follow-up Study; hs-CRP, high-sensitivity C-reactive protein; LDL-C, low-density lipoprotein cholesterol; MESA, Multi-Ethnic Study of Atherosclerosis; NHANES, National Health and Nutrition Examination Survey; NHS, Nurses' Health Study; U.S., United States

Study (Author, Year)	Study Design	Population	Outcome Focus	Key Findings
Colantonio et al. (2016) [1]	Prospective cohort	U.S. adults (ARIC, REGARDS, Kaiser Permanente)	Serum lipids and CHD	Higher LDL-C and total cholesterol were associated with increased coronary heart disease risk.
Lin et al. (2016) [2]	Prospective cohort (MESA)	Multi-ethnic U.S. adults	LDL-C, hs-CRP and CHD	Elevated LDL-C combined with higher hs-CRP increased incident coronary heart disease risk.
Ahmed et al. (2016) [3]	Prospective cohort (MESA)	Multi-ethnic U.S. adults	HDL-C and cardiovascular outcomes	Low HDL-C independently predicted coronary heart disease, cardiovascular disease, and mortality.
Sayed et al. (2023) [4]	Cross-sectional (NHANES)	U.S. adults	LDL-C prevalence and treatment	Approximately one in five U.S. adults had elevated LDL-C, despite improvements in awareness and treatment.
Aggarwal et al. (2022) [5]	Cross-sectional (NHANES)	U.S. adults	Lipid trends	Lipid concentrations improved over time, although many adults remained above recommended cholesterol targets.
Loprinzi et al. (2016) [7]	Cross-sectional study (NHANES)	4,745 U.S. adults	Healthy lifestyle characteristics and cardiovascular biomarkers	A greater number of healthy lifestyle characteristics was associated with more favorable cardiovascular biomarkers, including total cholesterol, LDL cholesterol, HDL cholesterol, triglycerides, and inflammatory markers.
Li et al. (2018) [8]	Prospective cohort	U.S. adults (NHS & HPFS)	Healthy lifestyle and life expectancy	Healthy lifestyle factors significantly increased life expectancy and reduced cardiovascular mortality.
Li et al. (2020) [9]	Prospective cohort	U.S. adults (NHS & HPFS)	Lifestyle and chronic disease-free survival	Healthy lifestyles prolonged life free from cardiovascular disease, diabetes, and cancer.
Schreiner et al. (2016) [10]	Longitudinal cohort (CARDIA)	Biracial U.S. adults	Lipid trends	Favorable lifestyle changes contributed to long-term improvements in lipid profiles.
Haslam et al. (2022) [11]	Cross-sectional	U.S. adults	Sugar-sweetened beverages and lipids	Greater intake of sugar-sweetened beverages was associated with adverse lipoprotein profiles.
Shan et al. (2020) [12]	Prospective cohort	U.S. adults (NHS & HPFS)	Healthy dietary patterns	Mediterranean-style and other healthy dietary patterns reduced cardiovascular disease risk.
Steinbrink et al. (2024) [13]	Cross-sectional (NHANES)	U.S. adults	Physical activity and cardiometabolic health	Higher physical activity levels were associated with healthier lipid profiles and lower cardiometabolic risk.
Zhu et al. (2021) [14]	Cross-sectional study	32,832 U.S. adults aged ≥20 years (NHANES 2007-2018)	Cardiovascular health metrics and lipid-related risk factors	Improvements in smoking status, physical activity, and total cholesterol were observed over time, although other cardiovascular health indicators showed less favorable trends, highlighting the continued importance of lifestyle-related risk factor management.
Cheng et al. (2022) [15]	Cross-sectional	3,638 U.S. adults with cardiovascular disease (NHANES 2007-2018)	Physical activity and cardiovascular risk factors	Adherence to recommended physical activity levels was associated with improvements in several cardiovascular risk factors, including body mass index and triglyceride levels, supporting the role of lifestyle interventions in cardiovascular risk reduction.
Höchsmann et al. (2021) [16]	Cluster-randomized trial	803 U.S. adults with obesity receiving primary care	Lifestyle intervention and cardiometabolic risk factors	A 24-month lifestyle intervention implemented in primary care improved several cardiometabolic risk factors, including HDL cholesterol and metabolic syndrome severity, while supporting the role of lifestyle-based management in routine clinical practice.
Boucher et al. (2015) [17]	Prospective cohort	Biracial U.S. adults with prediabetes	Diet, physical activity, and dyslipidemia	Healthy dietary habits and leisure-time physical activity reduced dyslipidemia and metabolic risk.
Joshi et al. (2016) [18]	Prospective cohort	Jackson Heart Study & Framingham Offspring Study	Remnant cholesterol and CHD	Higher remnant cholesterol independently predicted coronary heart disease.
Xu et al. (2026) [22]	Cross-sectional (BRFSS)	U.S. adults	High cholesterol prevalence	Self-reported high cholesterol prevalence increased between 2019 and 2023.
Carroll et al. (2024) [23]	Cross-sectional (NHANES)	U.S. adults	Total and HDL cholesterol	National estimates showed persistent prevalence of elevated total cholesterol and low HDL-C.
MacDonald et al. (2022) [24]	Cross-sectional (NHANES)	U.S. statin users	Dietary supplements and cholesterol	Statin users taking dietary supplements had more favorable serum cholesterol profiles.
Hou et al. (2026) [25]	Prospective cohort (All of Us)	U.S. adults	Wearable-measured physical activity	Greater objectively measured physical activity reduced chronic disease risk.
Hou et al. (2024) [26]	Observational (All of Us)	U.S. adults	Physical activity and chronic disease	Wearable device data demonstrated inverse associations between physical activity and chronic disease risk.
Miedema et al. (2015) [27]	Prospective cohort (ARIC)	Older U.S. adults	Statin eligibility	Many eligible adults were not receiving lipid-lowering therapy despite guideline recommendations.
Satija et al. (2017) [28]	Prospective cohort	U.S. adults (NHS & HPFS)	Plant-based diets and CHD	Healthful plant-based diets significantly reduced coronary heart disease risk.
Smiley et al. (2019) [29]	Cross-sectional (NHANES)	U.S. adults	Sleep duration and lipid profile	Adequate sleep duration was associated with higher HDL-C and lower triglyceride levels.
Jain & Ducatman (2018) [30]	Cross-sectional (NHANES)	U.S. adults ≥20 years	Smoking and lipid concentrations	Smoking was associated with lower HDL-C and higher triglyceride concentrations.

Table 3 presents the methodological quality assessment of the included observational studies using the NOS [20].

**Table 3:** Quality assessment for observational studies (Newcastle–Ottawa Scale)

Study (Author, Year)	Selection Score (Maximum 4)	Comparability Score (Maximum 2)	Outcome Score (Maximum 3)	Total Score (Maximum 9)	Quality Rating
Colantonio et al. (2016) [1]	4	2	3	9	High
Lin et al. (2016) [2]	4	2	3	9	High
Ahmed et al. (2016) [3]	3	2	2	7	Moderate
Sayed et al. (2023) [4]	4	2	3	9	High
Aggarwal et al. (2022) [5]	4	2	3	9	High
Loprinzi et al. (2016) [7]	4	2	3	9	High
Li et al. (2018) [8]	4	2	3	9	High
Li et al. (2020) [9]	4	2	3	9	High
Schreiner et al. (2016) [10]	4	2	3	9	High
Haslam et al. (2022) [11]	3	2	3	8	High
Shan et al. (2020) [12]	4	2	3	9	High
Steinbrink et al. (2024) [13]	3	2	3	8	High
Zhu et al. (2021) [14]	3	1	2	6	Moderate
Cheng et al. (2022) [15]	3	2	2	7	Moderate
Höchsmann et al. (2021) [16]	4	2	3	9	High
Boucher et al. (2015) [17]	4	2	3	9	High
Joshi et al. (2016) [18]	4	2	3	9	High
Xu et al. (2026) [22]	3	2	3	8	High
Carroll et al. (2024) [23]	3	2	3	8	High
MacDonald et al. (2022) [24]	3	2	3	8	High
Hou et al. (2026) [25]	4	2	3	9	High
Hou et al. (2024) [26]	3	2	2	7	Moderate
Miedema et al. (2015) [27]	4	2	3	9	High
Satija et al. (2017) [28]	4	2	3	9	High
Smiley et al. (2019) [29]	3	2	2	7	Moderate
Jain & Ducatman (2018) [30]	3	2	2	7	Moderate

Study Findings

The findings of this systematic review suggest that lifestyle medicine is associated with improved lipid profiles and favorable cardiovascular health outcomes among adults. The reviewed evidence indicates that lifestyle-based interventions may contribute to better lipid management and cardiovascular risk reduction; however, the observational nature of the included studies does not permit causal inferences [1,2]. Elevation of LDL-C, low HDL-C, elevation of triglycerides, and total cholesterol were regularly pointed out in all studies as the foremost modifiable risk factors for coronary heart disease, stroke, and other cardiovascular conditions [1-5]. Despite improvements in cholesterol management resulting from increased awareness and the expanded use of lipid-lowering therapies, uncontrolled dyslipidemia remains an important public health concern in the United States. Recent estimates indicate that the prevalence of self-reported high blood cholesterol increased from 11.3% in 2019 to 13.3% in 2023, highlighting the continued need for evidence-based lifestyle interventions as part of comprehensive cardiovascular risk management [4,5,21,23].

Numerous studies have consistently shown that the incidence of CVD and other chronic diseases can be significantly reduced through adherence to healthy lifestyle habits and practices. Following healthy dietary patterns, such as the Mediterranean diet or plant-based diets, and consuming foods rich in whole grains, legumes, and soluble fiber are associated with lower LDL-C levels and a reduced risk of CVD [12]. In addition, emerging pharmacological approaches, including newer weight-reduction therapies and contemporary antidiabetic treatments, may provide complementary benefits for lipid management and cardiometabolic risk reduction when used alongside evidence-based lifestyle interventions [4,5]. Similarly, engaging in regular aerobic and resistance exercise is associated with favorable changes in lipid profiles and improved cardiovascular health [12,16]. Current recommendations support at least 150 minutes of moderate-intensity aerobic activity or 75 minutes of vigorous-intensity aerobic activity per week, together with muscle-strengthening activities involving major muscle groups on at least two days per week. Regular physical activity contributes to lower triglyceride levels, improved overall cardiovascular health, and better long-term cardiometabolic outcomes [13,17]. It seems that the benefits of following healthy lifestyle practices go beyond just cardiovascular health [24,25].

Importantly, recent studies have shed more light on the importance of integrating lifestyle medicine into cardiovascular care. It has been shown that people who engage in more physical activity and are physically active on a daily basis, therefore achieving a higher physical activity status, have lower chances of suffering from chronic illnesses [26,27]. These findings highlight the potential value of wearable devices for objectively measuring physical activity and monitoring health behaviors, which may support future strategies aimed at improving cardiovascular health and reducing chronic disease risk [26]. Furthermore, previous studies have identified a substantial treatment gap among older adults who are eligible for statin therapy. A substantial proportion of eligible patients either do not receive lipid-lowering therapy or remain undertreated. Potential contributing factors include poor medication adherence, concerns about adverse effects, limited access to healthcare services, socioeconomic barriers, and variations in clinical practice patterns. These findings highlight an important opportunity to incorporate lifestyle-based strategies alongside pharmacological approaches to improve lipid management and cardiovascular risk reduction [27]. Furthermore, the shift in one's lifestyle towards healthful plant-based foods could lead to lower chances of developing coronary heart disease [28].

Although there is significant evidence to support lifestyle medicine, long-term compliance is a significant challenge. Poor diet quality and less favorable lipid profiles were consistently correlated with socioeconomic status, educational status, food insecurity, limited access to healthy foods, and unhealthy dietary behaviors [11,14,15]. Moreover, poor sleep quality and cigarette smoking were linked to decreased HDL-C and increased triglyceride levels and cardiovascular risk [29,30]. The results suggest that the success of cholesterol reduction requires individual behavior, as well as social and environmental factors that influence health.

Overall, multi-disciplinary lifestyle interventions with dietary counselling and structured physical activity, behavioral coaching and ongoing follow-up have the highest potential to achieve sustained cholesterol-lowering [16]. Therefore, lifestyle medicine should continue to be the foundation of dyslipidemia prevention and treatment, in addition to the use of pharmaceuticals when required and to maintain long-term cardiovascular health in adults [8,9].

Discussion

Lifestyle Medicine and Cholesterol Reduction

This systematic review illustrates that lifestyle medicine can effectively be utilized in the treatment of lipid levels and help reduce the risk of CVDs, especially among adults. High levels of LDL-C, total cholesterol, and triglycerides, together with low levels of HDL-C, are regarded as important risk factors that can be modified in patients with atherosclerosis, coronary artery disease, and stroke [1-5]. Even though statin medications help patients have better lipid control in the United States, some patients live with unmanageable dyslipidemia, which proves the need for complementary preventive measures [4,5]. Lifestyle modification remains a fundamental component of dyslipidemia management and is recommended as part of a comprehensive cardiovascular risk reduction strategy. Decisions regarding pharmacological therapy should be individualized and based on factors such as atherosclerotic CVD risk, LDL-C levels, diabetes status, established CVD, and other risk-enhancing factors [6].

The study showed that lifestyle medicine has become more important, as it can treat not only discomfort but also many cardiovascular risk factors. Lifestyle medicine encompasses several evidence-based interventions, including healthy dietary patterns, regular physical activity, weight management, smoking cessation, adequate sleep, and stress management. These strategies complement lipid-lowering medications and contribute to comprehensive cardiovascular risk reduction [8,9]. The researched issues revealed that among people who changed multiple aspects of their lifestyle, diseases were registered less often within a specific period, and people lived longer than those with unhealthy lifestyles [8,9]. Thus, it is necessary to consider lifestyle medicine as a basis for the treatment of patients with problems concerning cardiovascular health.

Dietary and Lifestyle Determinants of Lipid Profiles

This review highlighted dietary modification as one of the most effective non-pharmacological ways of reducing cholesterol levels. Different healthy dietary patterns such as the Mediterranean diet, plant-based diets and those rich in fruits, vegetables, legumes, nuts, whole grains and soluble fiber were shown to be associated with low concentrations of LDL-C and low risk of CVD [11,12,28]. However, unhealthy eating habits that include excessive intake of sugar-sweetened beverages and processed food were closely linked to a negative lipid profile and increased cardiovascular risk [11]. This finding is consistent with recent findings, which show that the quality of diet is one of the most important determinants of cholesterol control and cardiovascular health [12].

Other lifestyle factors were found to be important in cholesterol management and lipid profile improvement. Engaging in intensive aerobic and resistance exercise intervention resulted in high HDL-C, low triglycerides, improved insulin sensitivity, and ideal body weight [13,17,24]. In addition, adequate sleep duration contributed to the favorable lipid profiles, and smoking resulted in low HDL-C levels and high triglyceride concentrations [29,30]. Therefore, these findings suggest that multiple lifestyle practices, rather than a single intervention, are associated with favorable lipid profiles and support the multidimensional approach promoted by lifestyle medicine. However, lipid management should be individualized in specific populations, including patients with familial dyslipidemia syndromes and those receiving medications known to affect lipid metabolism, such as atypical antipsychotics, corticosteroids, retinoids, certain antihypertensive agents, and some antiretroviral therapies. In these populations, lifestyle interventions should be integrated with appropriate pharmacological management and individualized cardiovascular risk assessment.

Consistent findings have demonstrated that saturated fat intake and the extent of food processing have a significant influence on LDL-C levels and CVD [12,13]. Additionally, serum levels of HDL and triglycerides are adversely affected among smokers [30]. As a result, clinicians must set realistic expectations for patients that are specific, measurable, achievable, relevant and time-bound. Clinicians should also recommend comprehensive lifestyle modifications such as a diet rich in vegetables and greens, whole grains and soluble fiber; regular aerobic exercise and smoking cessation, tailored to every patient according to their needs

Barriers to Lifestyle Adherence and Future Clinical Practice

Though there is strong evidence supporting lifestyle medicine, long-term adherence to this approach remains a serious challenge for many individuals. The review revealed that socioeconomic status, educational background, availability of healthy foods, food insecurity, and health literacy were the main factors affecting the capability to change lifestyle behavior. In addition, adherence to lifestyle changes is also determined by motivation, behavior patterns, and the environment, which may prevent people from experiencing the full benefits of cholesterol-lowering therapies in terms of their long-term effectiveness [14,15].

The research indicates that successful implementation of lifestyle medicine requires much more than simple provision of health information. Organizations have to implement a team-based approach to health care delivery, including consultations with doctors, nutritionists, exercise specialists, and counselors, as well as individually tailored programs [16]. The application of new technologies such as remote monitoring systems and activity trackers will provide a novel and effective approach to achieving long-term lifestyle changes [25,26]. This means that it is crucial for clinical practice to combine evidence-based preventive lifestyle medicine with multidisciplinary techniques and digital health technologies so that the preventive effect of cholesterol-lowering may be achieved.

Limitations

This systematic review possesses certain limitations. First, the studies included in it were observational studies, making it not robust enough to draw conclusions. Second, only studies conducted in the United States have been included. Third, since lifestyle assessment methods and ways of assessing lipids differ among studies, the results vary substantially among the studies in the review. Lastly, there may be potential publication bias and reduction in generalizability due to exclusion of non-English studies.

## Conclusions

This systematic review finds that lifestyle medicine is a very effective non-pharmaceutical way of lowering cholesterol and enhancing the lipid profile in adults. Among lifestyle approaches that work are healthful dietary habits, regular exercise, weight control, smoking cessation, proper sleep, and stress management techniques that provide positive effects in the form of reduced risk of dyslipidemia. Nevertheless, it appears that being adherent to lifestyle medicine may not be easy due to challenges associated with lifestyles, behaviors, and the environment in general.

## References

[1] Colantonio LD, Bittner V, Reynolds K (2016). Association of serum lipids and coronary heart disease in contemporary observational studies. Circulation.

[2] Lin GM, Liu K, Colangelo LA, Lakoski SG, Tracy RP, Greenland P (2016). Low-density lipoprotein cholesterol concentrations and association of high-sensitivity c-reactive protein concentrations with incident coronary heart disease in the multi-ethnic study of atherosclerosis. Am J Epidemiol.

[3] Ahmed HM, Miller M, Nasir K, McEvoy JW, Herrington D, Blumenthal RS, Blaha MJ (2016). Primary low level of high-density lipoprotein cholesterol and risks of coronary heart disease, cardiovascular disease, and death: results from the multi-ethnic study of atherosclerosis. Am J Epidemiol.

[4] Sayed A, Navar AM, Slipczuk L, Ballantyne CM, Samad Z, Lavie CJ, Virani SS (2023). Prevalence, awareness, and treatment of elevated LDL cholesterol in US adults, 1999-2020. JAMA Cardiol.

[5] Aggarwal R, Bhatt DL, Rodriguez F, Yeh RW, Wadhera RK (2022). Trends in lipid concentrations and lipid control among US adults, 2007-2018. JAMA.

[6] Blumenthal RS, Morris PB, Gaudino M (2026). 2026 ACC/AHA/AACVPR/ABC/ACPM/ADA/AGS/APhA/ASPC/NLA/PCNA guideline on the management of dyslipidemia: a report of the American College of Cardiology/American Heart Association Joint Committee on Clinical Practice Guidelines. J Am Coll Cardiol.

[7] Loprinzi PD, Branscum A, Hanks J, Smit E (2016). Healthy lifestyle characteristics and their joint association with cardiovascular disease biomarkers in US adults. Mayo Clin Proc.

[8] Li Y, Pan A, Wang DD (2018). Impact of healthy lifestyle factors on life expectancies in the US population. Circulation.

[9] Li Y, Schoufour J, Wang DD (2020). Healthy lifestyle and life expectancy free of cancer, cardiovascular disease, and type 2 diabetes: prospective cohort study. BMJ.

[10] Schreiner PJ, Jacobs DR Jr, Wong ND, Kiefe CI (2016). Twenty-five year secular trends in lipids and modifiable risk factors in a population-based biracial cohort: the coronary artery risk development in young adults (CARDIA) Study, 1985-2011. J Am Heart Assoc.

[11] Haslam DE, Chasman DI, Peloso GM (2022). Sugar-sweetened beverage consumption and plasma lipoprotein cholesterol, apolipoprotein, and lipoprotein particle size concentrations in US adults. J Nutr.

[12] Shan Z, Li Y, Baden MY (2020). Association between healthy eating patterns and risk of cardiovascular disease. JAMA Intern Med.

[13] Steinbrink GM, Martinez J, Swartz AM, Cho CC, Strath SJ (2024). Is all physical activity equal? Investigating the total and domain-specific relationships between physical activity and cardiometabolic health in U.S. adults (NHANES 2013-2018). BMC Public Health.

[14] Zhu Z, Bundy JD, Mills KT (2021). Secular trends in cardiovascular health in US adults (from NHANES 2007 to 2018). Am J Cardiol.

[15] Cheng Y, Mou L, Li Z (2022). Trends in adherence to recommended physical activity and its association with cardiovascular risk factors in US adults with cardiovascular disease: a cross-sectional study. BMC Cardiovasc Disord.

[16] Höchsmann C, Dorling JL, Martin CK (2021). Effects of a 2-year primary care lifestyle intervention on cardiometabolic risk factors: a cluster-randomized trial. Circulation.

[17] Boucher AB, Adesanya EA, Owei I (2015). Dietary habits and leisure-time physical activity in relation to adiposity, dyslipidemia, and incident dysglycemia in the pathobiology of prediabetes in a biracial cohort study. Metabolism.

[18] Joshi PH, Khokhar AA, Massaro JM (2016). Remnant lipoprotein cholesterol and incident coronary heart disease: the Jackson Heart and Framingham Offspring cohort studies. J Am Heart Assoc.

[19] Page MJ, McKenzie JE, Bossuyt PM (2021). The PRISMA 2020 statement: an updated guideline for reporting systematic reviews. BMJ.

[20] Schardt C, Adams MB, Owens T, Keitz S, Fontelo P (2007). Utilization of the PICO framework to improve searching PubMed for clinical questions. BMC Med Inform Decis Mak.

[21] Carra MC, Romandini P, Romandini M (2025). Risk of bias evaluation of cross-sectional studies: adaptation of the Newcastle-Ottawa scale. J Periodontal Res.

[22] Xu K, Pankowska MM, Sekkarie A, Imoisili OE, Loustalot F (2026). Increase in prevalence of self-reported high blood cholesterol among adults, United States, 2019-2023. Prev Chronic Dis.

[23] Carroll MD, Fryar CD, Gwira JA, Iniguez M (2024). Total and high-density lipoprotein cholesterol in adults: United States, August 2021-August 2023. NCHS Data Brief.

[24] MacDonald TS, Davis KE, Tucker WJ, Miketinas DC (2023). Serum cholesterol differences between statin users who take dietary supplements and those who do not: NHANEs 2013-2018. Curr Dev Nutr.

[25] Hou Y, Cui E, Lim K, Chow LS, Howell M, Ikramuddin S, Zhang R (2026). Association of chronic disease risk and physical activity measured by wearable devices in the All of Us program. Commun Med (Lond).

[26] Hou Y, Cui E, Ikramuddin S, Zhang R (2024). Association of physical activity from wearable devices and chronic disease risk: insights from the All of Us research program (PREPRINT). medRxiv.

[27] Miedema MD, Lopez FL, Blaha MJ, Virani SS, Coresh J, Ballantyne CM, Folsom AR (2015). Eligibility for statin therapy according to new cholesterol guidelines and prevalent use of medication to lower lipid levels in an older US Cohort: the Atherosclerosis Risk in Communities Study Cohort. JAMA Intern Med.

[28] Satija A, Bhupathiraju SN, Spiegelman D (2017). Healthful and unhealthful plant-based diets and the risk of coronary heart disease in U.S. adults. J Am Coll Cardiol.

[29] Smiley A, King D, Harezlak J, Dinh P, Bidulescu A (2019). The association between sleep duration and lipid profiles: the NHANES 2013-2014. J Diabetes Metab Disord.

[30] Jain RB, Ducatman A (2018). Associations between smoking and lipid/lipoprotein concentrations among US adults aged ≥20 years. J Circ Biomark.

